# Joint distribution of child mortality and wealth across 30 sub-Saharan African countries over 2000-2019

**DOI:** 10.7189/jogh.13.04009

**Published:** 2023-02-24

**Authors:** Ryoko Sato, Sarah Bolongaita, Solomon Tessema Memirie, Kenneth Harttgen, Jan-Walter De Neve, Stéphane Verguet

**Affiliations:** 1 Department of Global Health and Population, Harvard T.H. Chan School of Public Health, Boston, Massachusetts, USA; 2 Department of Pediatrics and Child Health, College of Health Sciences, Addis Ababa University, Addis Ababa, Ethiopia; 3 Department of Humanities, Social and Political Sciences, ETH Zurich, Zurich, Switzerland; 4 Heidelberg Institute of Global Health, Heidelberg University, Heidelberg, Germany

## Abstract

**Background:**

While reductions in child mortality have been observed across sub-Saharan African countries in the last 30 years, narrowing the gaps in under-five mortality across socioeconomic groups also requires an understanding of the multiple associations between health and welfare and socioeconomic drivers. We examined the probability density distributions in under-five mortality within countries and joint pathways of under-five mortality and wealth over time.

**Methods:**

We used 69 Demographic and Health Surveys and 19 Malaria Indicator Surveys from 30 sub-Saharan African countries, with each country having at least two surveys conducted since 2000. We constructed a cross-country wealth index and estimated under-five death prevalence. We examined the pure distribution in under-five mortality prevalence and the joint probability distribution of wealth and under-five mortality prevalence over time, including the area of confidence ellipse which spanned the two dimensions of mortality and wealth and covered 75% of the mass of the joint distribution.

**Results:**

Most countries experienced decreases in under-five mortality along with increases in wealth over time. However, we observed great variations in the evolution of the joint distributions across countries over time. For instance, the areas of confidence ellipse ranged from 0.178 in Ethiopia (2000) to 1.119 in Angola (2006). The change (over time) in the area of confidence ellipses ranged from 0.010 in Tanzania to 0.844 in Angola between the 2000s and 2010s. The ranking of country performance on under-five mortality varied greatly, depending on whether performance summary indicators were based on disaggregation by wealth or on full non-disaggregated distributions.

**Conclusions:**

Our analysis points to the relevance of full distributions of health and joint distributions of health and wealth as complementary indicators of distributions of health across socioeconomic status, in assessing country performance on health.

Child mortality decreased remarkably over the last three decades, especially in low- and middle-income countries (LMICs) [1]. While the global under-five mortality rate dropped from 93 per 1000 live births in 1990 to 38 per 1000 in 2019, it was reduced from 178 per 1000 in 1990 to 78 per 1000 in 2019 in sub-Saharan Africa (SSA) [1,2]. Besides SSA facing much higher child mortality than other regions, these large reductions mask great heterogeneity across and within SSA countries, particularly along the socioeconomic gradient [3]. According to the 2018 Nigeria Demographic and Health Survey (DHS), under-five mortality would vary from 173 per 1000 among households in the country’s lowest wealth quintile to 53 per 1000 among households in the highest wealth quintile; and from 30 per 1000 in Ogun state (South West) to 213 per 1000 in Jigawa state (North West) [4].

The documented major overall reductions in under-five mortality can be attributed to multiple factors. Importantly, the strong momentum toward the Millennium Development Goals (MDGs) has been accompanied by the scale-up of high-impact programs, including the scaling up of immunization coverage and the expansion of primary health care [5-7]. Beyond the health sector, social determinants (like education, women empowerment and economic development) have likely played a significant role [8-12]. However, while large improvements in under-five mortality and intervention coverage have been observed, significant gaps between the poor and the rich have remained [13,14].

Narrowing the gaps in under-five mortality across socioeconomic groups within LMICs requires an understanding of not only the health, intersectoral, and social determinants of child mortality, but also of the multilateral associations between health and socioeconomic drivers (like material welfare, including income and education) [15]. This necessitates the investigation of how income and health are jointly produced and may interact bilaterally and an examination of the full extent of the distribution of health within countries, regardless of its disaggregation across socioeconomic groups.

We studied the evolution of under-five mortality since 2000 in sub-Saharan Africa, the world region with the highest and most inequal child mortality rates [1]. Specifically, we drew from 88 DHS and Malaria Indicator Surveys [3] from 30 countries between 2000 and 2019. We aimed to study the joint probability distribution of under-five mortality and wealth across countries and time, and to identify whether specific “co-productions” could be stressed beyond the traditional determinants through varying levels of income. Although numerous previous studies have evaluated the socioeconomic distribution, across wealth quintiles, of under-five mortality in LMICs (e.g. Chao et al. 2018 [16]), to our knowledge, our paper is one of the first to systematically examine the joint distribution of under-five mortality and wealth over time, along with the probability density (full) distributions in under-five mortality, for a great number of countries and years in SSA.

## METHODS

### Data sources and sample population

We compiled DHS/MIHS from sub-Saharan African countries where two surveys were conducted in or after 2000 (e.g. one survey conducted in the 2000s and one in the 2010s) and which included data on the birth history of women and household assets. A key advantage of the DHS/MIS is that they provide nationally representative samples for many countries that are comparable and of consistent quality of reporting over time [17]. However, one disadvantage is that they do not include information on income or expenditure, thus our focus on wealth here. The resulting 88 country-years (covering 30 countries) substantially varied in geography and health and economic indicators across SSA (Table S1 in the **Online Supplementary Document**). We focused on SSA because the region still faces high mortality levels, despite experiencing substantial declines in child mortality over time [18]. For each survey, the birth recode files provided a full birth history of all women interviewed during the survey; they include data for the mother of each child [19]. The birth history data are typically collected from all women aged 15 to 49 years. We included all children born in a surveyed household (from the survey recode files) and for whom complete data on survival status and household assets (wealth) were available.

### Construction of comparable wealth scores across surveys

We used the assembled multiple country-year surveys to construct wealth scores that would be consistent and comparable across countries and survey years. While DHS/MIS data provide wealth scores for each country and survey year, such wealth scores are comparable only within the same country and survey year. Thus, to make the wealth scores comparable across all countries and survey years, we proceeded in four steps. First, we selected eight variables that indicated asset holdings of households which were common across all surveys, whether a household had: electricity; a TV; a refrigerator; a bicycle; a motorcycle; a car; a source of drinking water; and a type of toilet facility. Second, using these eight asset variables from all the DHS/MIS countries and survey years selected for the analysis, we computed a wealth score using principal component analysis [20]. Because we used the same asset variables and the same eigenvector across all countries and survey years, the resulting constructed wealth scores would be consistent and comparable across space and time. We calculated the wealth score for each cluster from each country-year survey to have sufficient variation.

### Computing under-five death prevalence

We calculated under-five death prevalence per the wealth subgroup computed in each country-year (i.e. each survey). We ranked clusters in each country-year according to its wealth score. For each cluster, we calculated an estimate of under-five death prevalence based on the birth records available in the survey. We defined death prevalence as the number of children who died at or before age five divided by the total number of children under age five.

### Distributions of under-five death prevalence

As an input for our analysis on joint distributions, we reported the marginal probability density distribution of under-five death prevalence in each wealth level across country-years. We then extracted summary indicators of mean and dispersion, including cumulative distributions. We also examined the difference across the 10^th^ and 90^th^ percentiles of the probability density (full) distribution in under-five death prevalence for each country-year.

### Joint distribution of under-five death prevalence and wealth

For each country-year, we could count the number of children who died at or before age five (i.e. under-five deaths) out of the total number of children who were born in a given wealth subgroup. Consequently, we could construct a 2D plane constituted of grids (along wealth levels and levels of under-five death prevalence) to derive a joint probability distribution of under-five death prevalence and wealth. This bivariate probability distribution defined the “simultaneous behaviour” of two conditionally dependent variables: a continuous variable for mortality (X) and a discrete variable for wealth (Y). From this joint distribution, we could extract 75% probability ellipses (i.e. containing 75% of the points in the plane) including means and standard deviations that summarize the 2D distribution of mortality and wealth.

### Comparing full and joint distributions

Lastly, we examined the correlation between indicators of full distributions of mortality (i.e. from death prevalence levels across 10^th^ and 90^th^ percentiles) and of wealth gradients of mortality (i.e. from death prevalence levels across 1st and 5th wealth quintiles).

All analyses were conducted using STATA (version 15) and R software (www.r-project.org, version 3.6.0).

## RESULTS

### Full distributions

**Figure 1:**
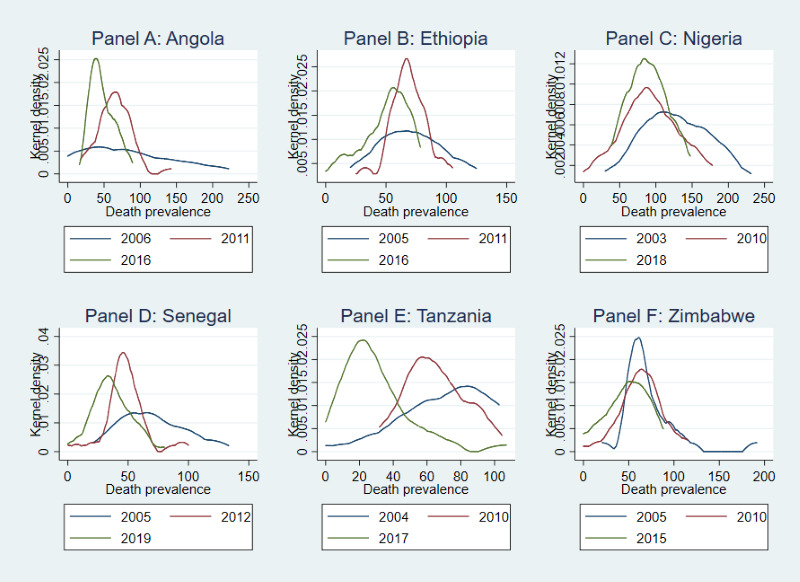
Probability density (full) distribution of under-five death prevalence (per 1000), 2004-2019, for six sub-Saharan African countries. **Panel A:** Angola. **Panel B:** Ethiopia. **Panel C:** Nigeria, **Panel D:** Senegal. **Panel E:** Tanzania. **Panel F:** Zimbabwe.

The evolution of the full distributions in under-five death prevalence over time for six countries is presented in **Figure 1**. This selection of countries is meant to capture geographical variety and heterogeneity within SSA and includes: Angola (Central), Ethiopia (Eastern), Nigeria (Western), Senegal (Western), Tanzania (Eastern), and Zimbabwe (Southern). Overall, the death prevalence estimates shifted left (toward mortality reduction) over time. All countries, with the notable exception of Zimbabwe, presented wider distributions (in death prevalence) in earlier years which thinned over time (see also Figure S1 in the **Online Supplementary Document** for cumulative distributions).

**Figure 2:**
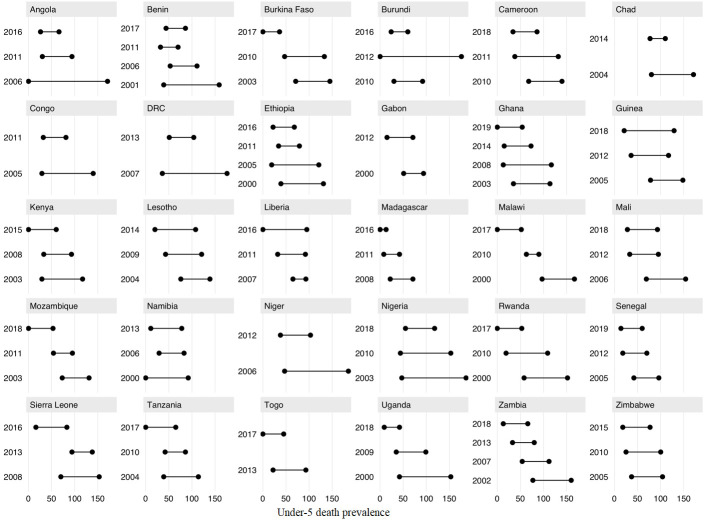
Evolution of the extent of under-five death prevalence (per 1000) across the 10th to 90th percentiles of the probability density (full) distribution of under-five death prevalence, across all country-years.

To characterize and summarize these full distributions in mortality, we examined the difference in death prevalence between the 10^th^ and 90^th^ percentiles (of the distribution) for each country-year (**Figure 2**). The difference over time across 10^th^ and 90^th^ percentiles decreased in countries like Angola, Chad, the Democratic Republic of Congo, Madagascar, Niger, Nigeria, and Uganda (**Figure 2**), but increased in countries such as Gabon, Guinea, Lesotho, and Liberia. We saw substantial variations across countries when ranking them based on death prevalence at the 90^th^ percentile compared to death prevalence at the 10^th^ percentile (Figure S2 in the **Online Supplementary Document**). For instance, Burundi (2012) had one of the highest death prevalence among the 90^th^ percentile, but the lowest one among the 10^th^ percentile. Malawi had much closer death prevalences accross the 90^th^ and 10^th^ percentiles. All percentiles in the probability density distributions of under-five death prevalence across all country-years, along with an evolution of the ratio in under-five death prevalence across 90^th^ and 10^th^ percentiles are available in Table S2 and Figure S5 in the **Online Supplementary Document**.

### Joint distributions

**Figure 3:**
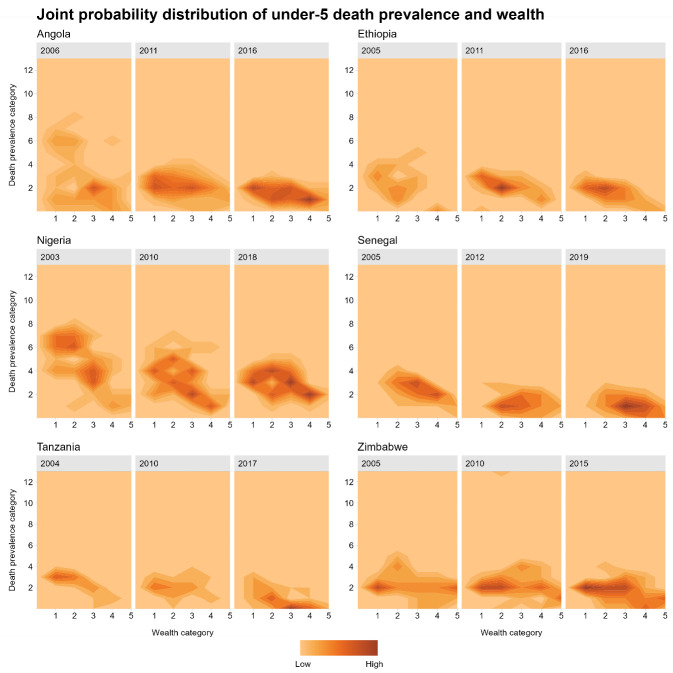
Joint probability density distribution of under-five death prevalence and wealth, 2004-2019, for six sub-Saharan African countries: Angola (2006, 2011, 2016), Ethiopia (2005, 2011, 2016), Nigeria (2003, 2010, 2018), Senegal (2005, 2012, 2019), Tanzania (2004, 2010, 2017), Zimbabwe (2005, 2010, 2015). The x-axis indicates the wealth category which varies from 1 (poorest) to 5 (richest). We calculated the wealth scores per country and per year using standardized measurements to allow comparisons across country-years and divided all country-years by five wealth categories equally. The y-axis indicates the category for the under-five death prevalence: the number of children who died at or before age five divided by the total number of children under age five multiplied by 1000/25 (division by 25 is used for scaling purposes).

The evolution of the joint distribution of under-five death prevalence and wealth over time for our selection of six countries is presented in **Figure 3**, while summary indicators of means and standard deviations as well as on area of confidence ellipses for these joint distributions are available in **Table 1**. Death prevalence decreased and wealth levels increased over time for all six countries. Confidence ellipses (**Table 1** and Figure S3 in the **Online Supplementary Document**) indicate how the joint distributions span over the two dimensions of mortality and wealth. For example, Angola substantially reduced the spread of the distribution of mortality over time, from 1.12 to 0.28 regarding the area of confidence ellipse (measured in death prevalence by wealth units); the spread did not change over time for other countries such as Senegal, Tanzania, and Zimbabwe, while increases were observed in Ethiopia. Simultaneously among all 30 countries, overall death prevalence decreased, while wealth increased (**Figure 3** and Figure S4 in the **Online Supplementary Document**).

**Table 1:** Estimated two-dimensional means and standard deviations, with areas of confidence ellipse (75% confidence surfaces) along under-five death prevalence and wealth, for six sub-Saharan African countries over 2000-2019

Country year	Under-five death prevalence*		Wealth		Area of confidence ellipse
	**Mean**	**SD**	**Mean**	**SD**	
**Angola**					
2006	0.082	0.275	2.376	2.200	1.119
2011	0.064	0.245	2.581	2.251	0.424
2016	0.049	0.216	2.723	2.175	0.275
**Ethiopia**					
2000	0.121	0.326	1.032	1.090	0.178
2005	0.087	0.282	1.361	1.394	0.343
2011	0.073	0.259	1.704	1.523	0.258
2016	0.060	0.237	1.957	1.633	0.262
**Nigeria**					
2003	0.140	0.347	2.521	1.906	0.517
2008	0.100	0.300	2.706	1.817	0.620
2018	0.095	0.293	2.810	1.821	0.370
**Senegal**					
2005	0.077	0.266	2.701	1.874	0.283
2012	0.047	0.211	2.757	1.718	0.262
2019	0.037	0.189	3.464	1.884	0.246
**Tanzania**					
2004	0.083	0.276	1.521	1.050	0.305
2010	0.062	0.241	1.721	1.349	0.295
2017	0.030	0.170	2.274	1.618	0.332
**Zimbabwe**					
2005	0.071	0.256	2.546	2.019	0.526
2010	0.065	0.246	2.644	2.014	0.395
2015	0.053	0.224	3.072	2.224	0.381

SD – standard deviation

*Death prevalence is the number of children who died at or before age five, divided by the total number of children under age five.

### Comparing full and joint distributions

Subsequently, we compared indicators of full mortality distributions (i.e. death prevalence by decile) and indicators of wealth-disaggregated distributions of mortality (i.e. death prevalence by wealth quintile) (Figure S6 in the **Online Supplementary Document**). We observed a positive correlation between the two indicators in terms of absolute differences (i.e. prevalence in 90^th^ percentile minus prevalence in 10^th^ percentile). When we turned to ratios (i.e. prevalence in 90^th^ percentile divided by prevalence in 10^th^ percentile), similarly, we observed positive correlations. However, overall, we found large variations across survey country-years, especially regarding rankings. Pointedly, the correlation coefficients were around 0.53 for the ranks in difference indicators and 0.27 for the ranks in ratio indicators.

### Country rankings by performance indicator

**Figure 4:**
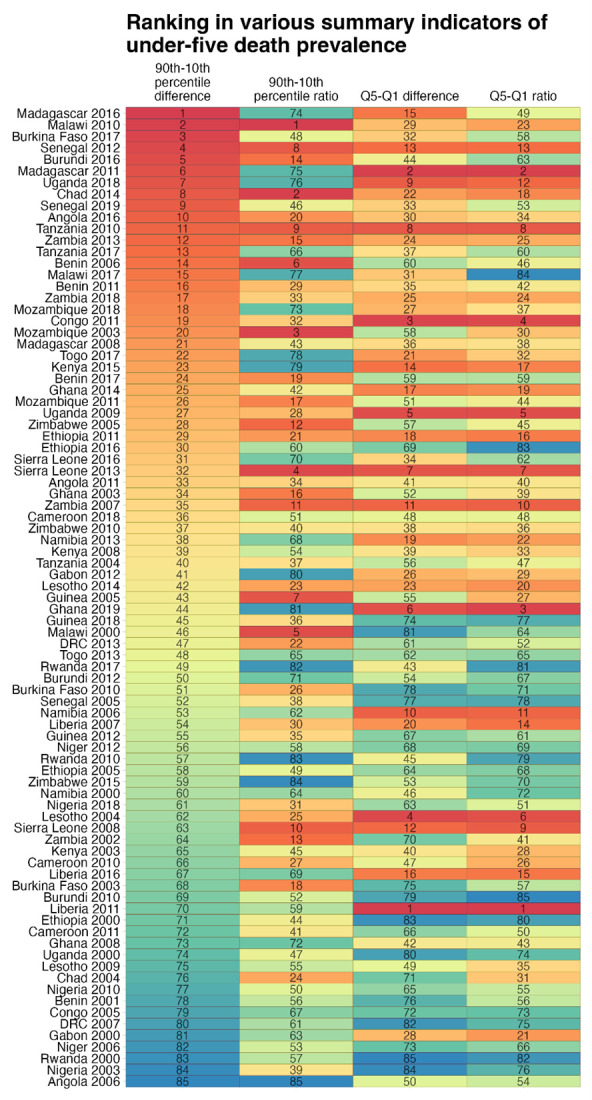
Ranking (across all country-years, since 2000 for 30 sub-Saharan African countries) in different summary indicators of distribution in under-five death prevalence. Each column indicates the ranking of the country-years based on the following summary indicators. Column 1: difference in under-five death prevalence between 90^th^ and 10^th^ percentiles. Column 2: ratio of under-five death prevalence in 90^th^ percentile divided by under-five death prevalence in 10^th^ percentile. Column 3: difference in under-five death prevalence between wealth quintiles 1 and 5. Column 4: ratio of under-five death prevalence in wealth quintile 1 divided by wealth quintile 5.

We found wide variations in performance indicators across countries and years. For example, the difference in under-five death prevalence between the poorest and richest quintiles was close to 20 in Gabon in 2000, while the difference between the 10^th^ and 90^th^ percentiles more than 40 in the same country. Likewise, Kenya’s under-five death prevalence did not differ significantly across wealth quintiles in 2015, while the difference between the 90^th^ and 10^th^ percentiles was over 50. This seemed to indicate the relevance of indicators characterizing full mortality distributions, as they appeared to point to complementary information to wealth gradients in mortality. This is demonstrated when proceeding to rankings of summary indicators across all country-years (**Figure 4**). For some countries, the rankings differed substantially depending on used indicators. For example, Burundi in 2016 was ranked fifth in terms of the difference between 10^th^ and 90^th^ percentiles of under-five death prevalence, while it was ranked 44^th^ regarding the difference between the poorest and richest quintiles of death prevalence. Likewise, Sierra Leone in 2008 was ranked 63 in terms of the difference between 10^th^ and 90^th^ percentiles, but ranked 12th in terms of the difference between the poorest and richest quintiles.

## DISCUSSION

We analyzed the joint distributions of health outcomes (via the lens of under-five death prevalence) and wealth (proxied by asset indices) across 30 sub-Saharan African countries between 2000 and 2019. We found that most countries experienced decreases in under-five mortality along with increases in wealth over time. Meanwhile, we observed great variations in the evolution of the joint distributions over time across countries.

When turning to the examination of full distributions of under-five deaths, we observed large variations across country-years. For example, in Liberia, under-five death prevalence within the 10^th^ percentile decreased substantially from 2007 to 2016, while its 90^th^ percentile did not vary much over the same time period; consequently, disparities increased over time in Liberia. Meanwhile, Uganda showed a contrasting pattern: under-five death prevalence decreased drastically among the 90^th^ percentile, as well as (although less so) in the 10^th^ percentile, leading to a decrease in disparities over time.

Overall, as expected, under-five death prevalence decreased simultaneously as wealth levels increased over time across all countries in our sample. This is consistent with observations already documented elsewhere [21]. However, we found great variations in the trajectories and joint distributions between child mortality and wealth across country-years. For example, death prevalence in Angola decreased substantially from 82 per 1000 in 2006 to 49 per 1000 in 2016, while wealth levels only modestly increased, resulting in a unidimensional evolution of the joint distribution over time. We also detected a similar evolution for Nigeria. Both countries are oil exporters and have been experiencing a recession since 2016 [22,23], which might explain the evolution of their wealth distributions [24]. Meanwhile, studies have also indicated reductions in inequalities in child health (such as wasting) in these countries [25]. However, Ethiopia observed a rather substantial decrease in death prevalence alongside a simultaneous substantial increase in wealth levels. Prior to the COVID-19 pandemic, Ethiopia had one of the fastest growing economies in the world since 2000 [26] while showing tremendous achievements in health outcomes such as prolonged life expectancy, reductions in malnutrition, and HIV prevalence [27]. Through this study, we found that joint pathways of health and wealth over time would overall be consistent across countries, yet the spread of these co-distributions could greatly vary in each country. Elucidating these differences may point to mutually reinforcing joint productions of health and wealth worthy of future research.

Although the ranking of countries in terms of mortality prevalence changed depending upon the indicator used, that is whether across mortality deciles or across wealth-quintile-specific mortality, overall, there were consistent correlations between rankings across the two indicators. However, significant differences appeared for some countries, pointing to the relevance of reporting on full distributions of under-five mortality, besides distributions of under-five mortality across socioeconomic groups (wealth quintiles in this study).

Nevertheless, our analysis has important limitations. First, in some clusters extracted from the DHS/MIS, the sample sizes might be too small to robustly and accurately calculate our measures of death prevalence for each wealth level constructed (Table S3 in the **Online Supplementary Document**). Therefore, our levels of under-five death prevalence should be interpreted with caution. Second, we only provide a description of the joint distributions of under-five mortality and wealth and of the full distributions of under-five death prevalence. This drastically restricts the analysis around a few selected indicators (under-five mortality and asset indices in this study), while many other indicators of health (e.g. adult mortality), wealth (e.g. income), and health services coverage (e.g. immunization rates) could be studied. Most importantly, our analysis does not elucidate causal mechanisms and potential explanations for why we observed small or large variations across country-years, like the paramount importance of the social determinants of health [28,29]. Third, we solely used simple summary indicators (e.g. confidence ellipses, 10-90^th^ percentile ranges) whereas a plethora of alternative indicators (e.g. coefficient of variation, Gini index) could have been implemented instead. Fourth, while we applied principal component analysis methods to construct wealth indices consistent across countries and years, future work should validate our methods, as our study is unique in attempting to derive wealth indices comparable across space and time [30,31].

## CONCLUSIONS

We aimed to preliminarily examine the full distributions of outcomes critically underlying the construction of summary development indicators like the human development index [32]. Rather than providing a definite and precise picture, we stress that examining joint distributions and full distributions in outcomes of interest, like distributions of incomes in economics [33], would be worthy of future explorations, especially in characterizing health disparities within LMICs.

## Additional material


Online Supplementary Document
