# Supplementary material for: Joint distribution of child mortality and wealth across 30 sub-Saharan African countries over 2000-2019
Source: J Glob Health. 2023 Feb 24;13:04009. doi: 10.7189/jogh.13.04009 (PMC9949559; doi:10.7189/jogh.13.04009)
Supplement: Online Supplementary Document [file jogh-13-04009-s001.pdf]

ONLINE SUPPLEMENTARY DOCUMENT

Title: Joint distribution of child mortality and wealth across 30 sub-Saharan African countries over 2000-2019

Authors: Ryoko Sato, Sarah Bolongaita, Solomon Tessema Memirie, Kenneth Harttgen, Jan-Walter De Neve, Stéphane Verguet

Supplementary webappendix

Figure S1. Cumulative probability density (full) distribution of under-five death prevalence (per 1,000), 2004-2019, for six sub-Saharan African countries: Angola (a), Ethiopia (b), Nigeria (c), Senegal (d), Tanzania (e), and Zimbabwe (f).

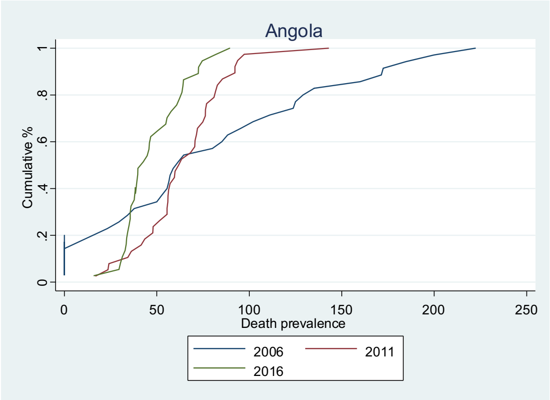

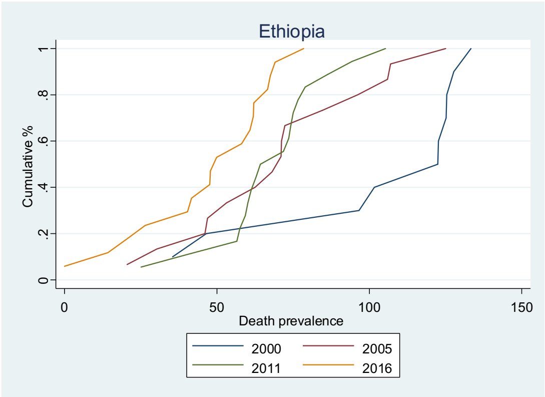

(a) (b)

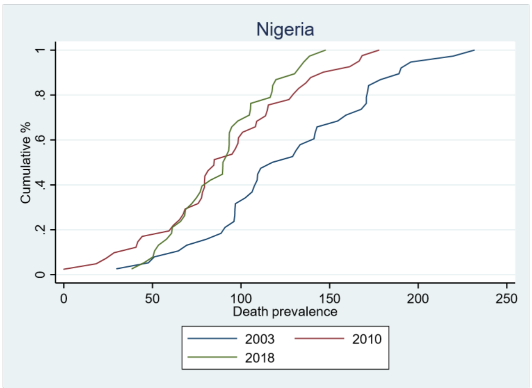

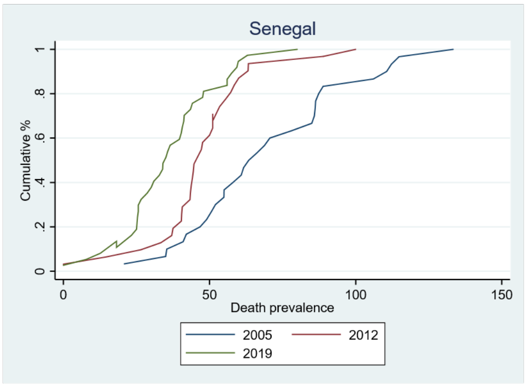

(c) (d)

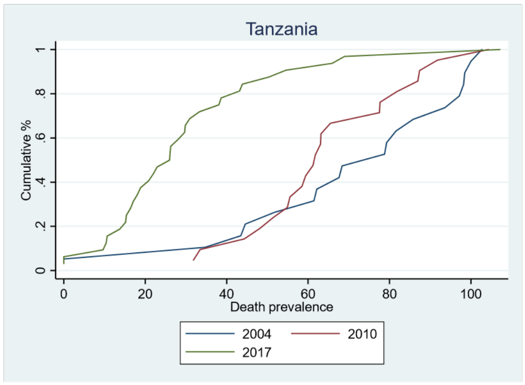

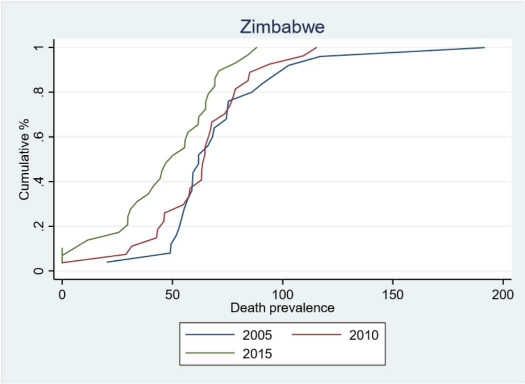

(e) (f)

Figure S2. Ranking of the extent of under-five death prevalence (per 1,000) across the 10th to 90th percentiles of the probability density ("pure") distribution of under-five death prevalence across all country-years.

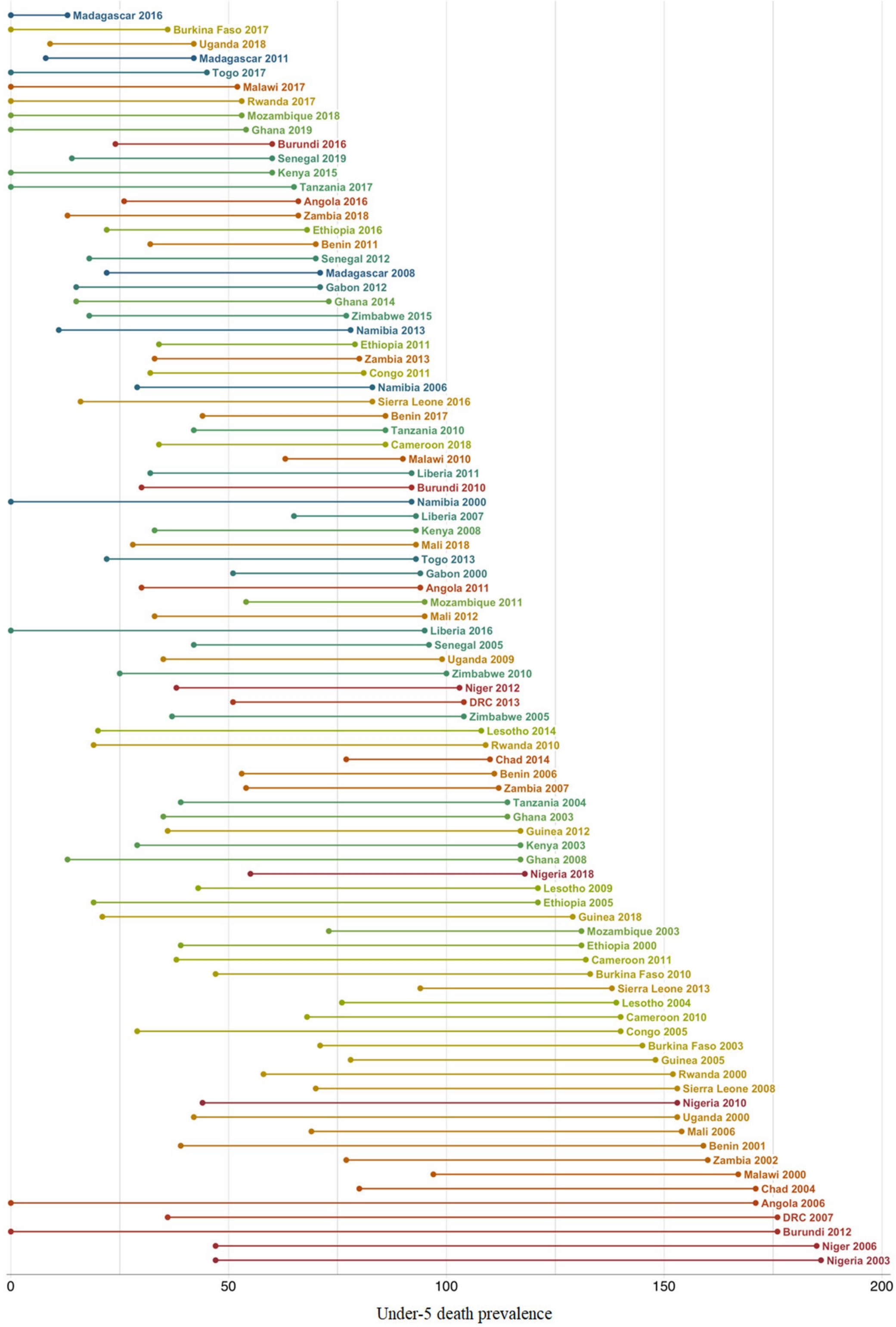

Figure S3. Uncertainty (characterized by 75% confidence ellipses) of joint probability density distribution of under-five death prevalence (per 1,000) and wealth, 2004-2019, for six sub-Saharan African countries: Angola (a), Ethiopia (b), Nigeria (c), Senegal (d), Tanzania (e), and Zimbabwe (f).

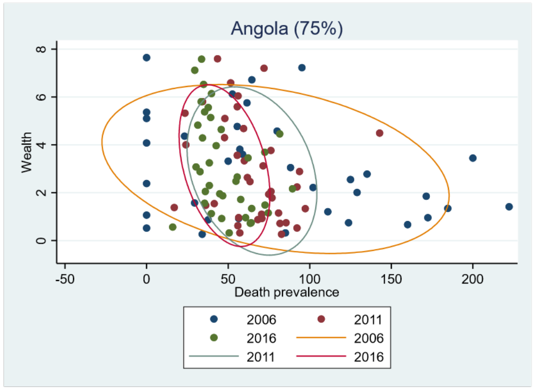

(a)

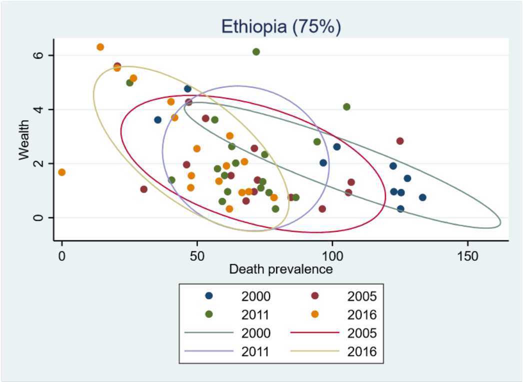

(b)

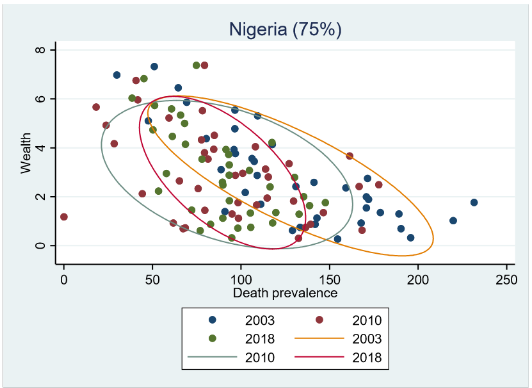

(c)

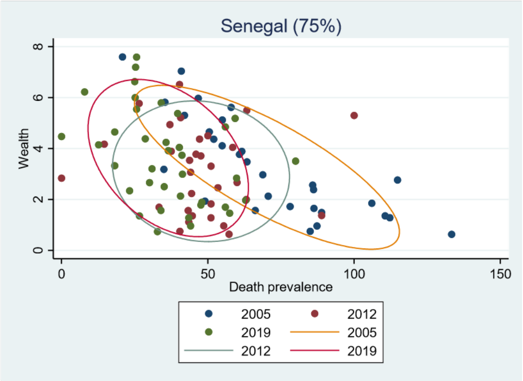

(d)

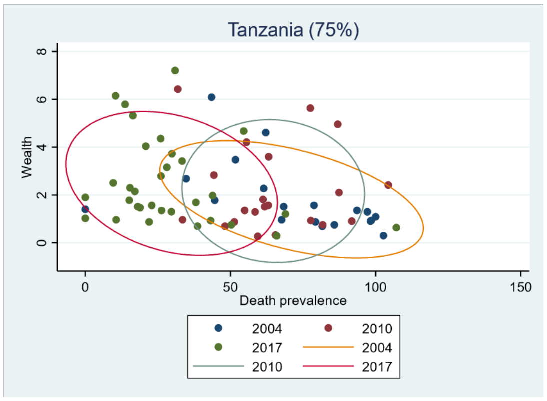

(e)

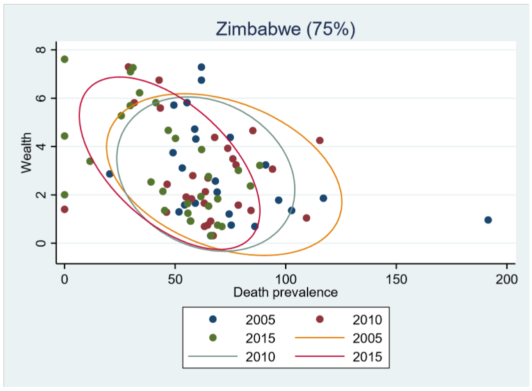

(f)

Figure S4. Evolution of the joint probability density distribution of under-five death prevalence and wealth since 2000 for 30 sub-Saharan African countries, based on data from the Demographic and Health Surveys and Malaria Indicator Surveys.

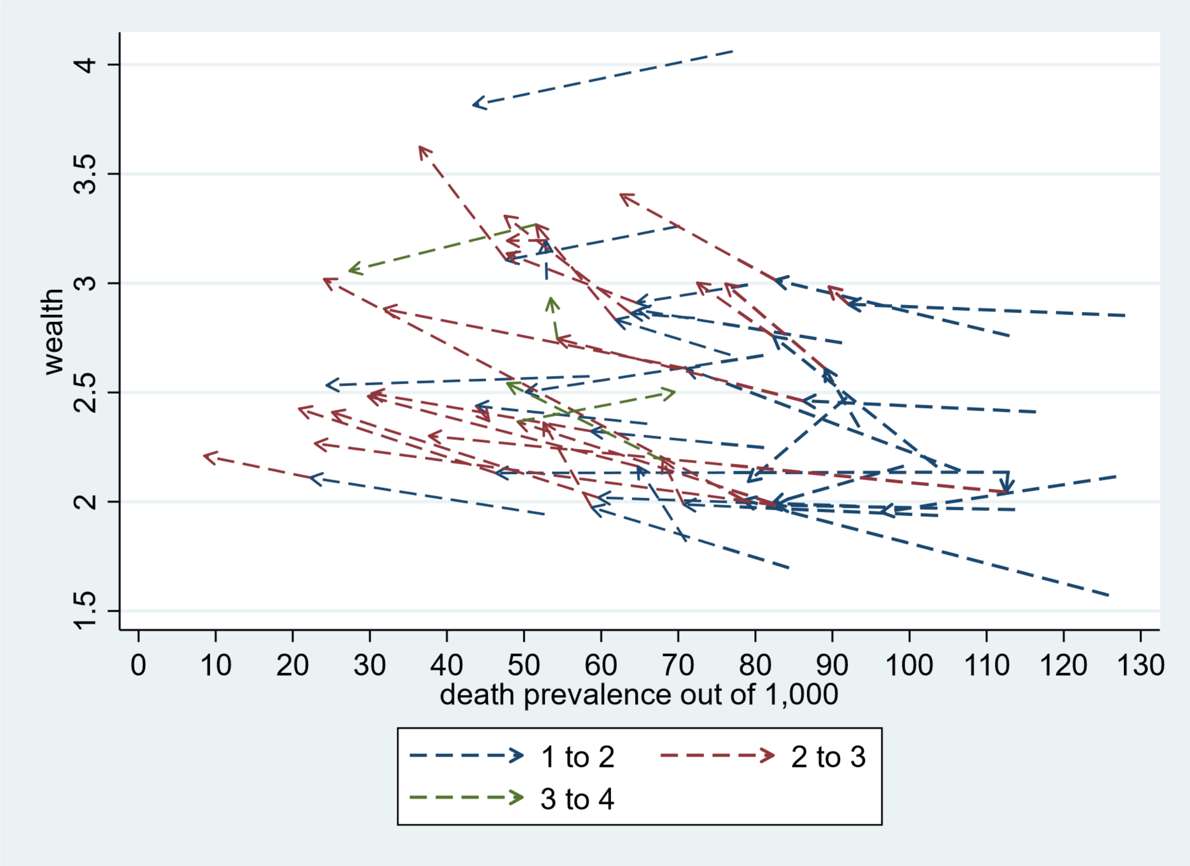

Note: 1 to 2 indicates the transition from the oldest survey to the 2nd oldest survey; 2 to 3 indicates the transition from the 2nd to the 3rd oldest survey (or, the latest survey if three surveys were available); 3 to 4 indicates the transition from the 3rd oldest to the 4th oldest survey (or, the latest survey if four surveys were available).

Figure S5. Ratio of under-five death prevalence across the 90th and 10th percentiles: earliest survey year (x-axis) and latest survey year (y-axis).

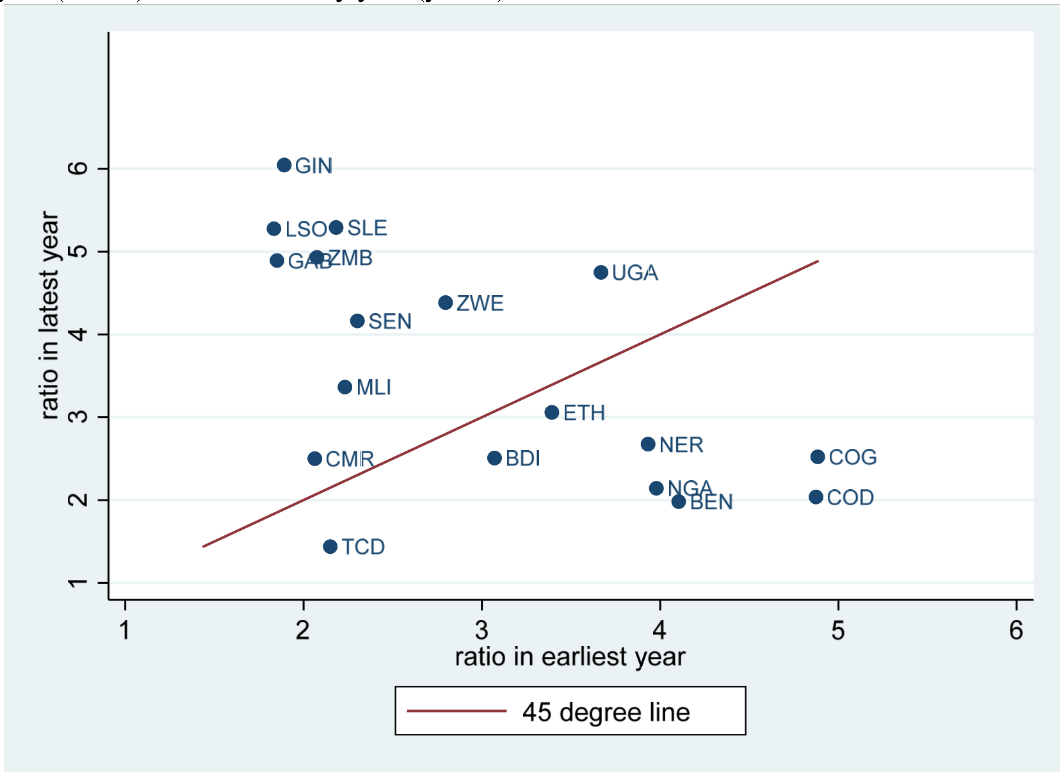

Notes: Countries that had an estimate of 0 for under-five death prevalence for the 10th percentile were excluded.
BEN=Benin; BDI=Burundi; CMR=Cameroon; TCD=Chad; COG=Congo; COD=DRC; ETH=Ethiopia; GAB=Gabon; GIN=Guinea; LSO=Lesotho; MLI=Mali; NER=Niger; NGA=Nigeria; SEN=Senegal; SLE=Sierra Leone; UGA=Uganda; ZMB=Zambia; ZWE=Zimbabwe.

Figure S6. Indicators of wealth gradients of mortality (i.e. drawing from the under-five death prevalence across the 1st and 5th wealth quintiles) compared with indicators of pure distributions of mortality (i.e. drawing from the under-five death prevalence across the 10th and 90th percentiles). Estimates extracted for 30 sub-Saharan African countries from 88 Demographic and Health Surveys and Malaria Indicator Surveys.

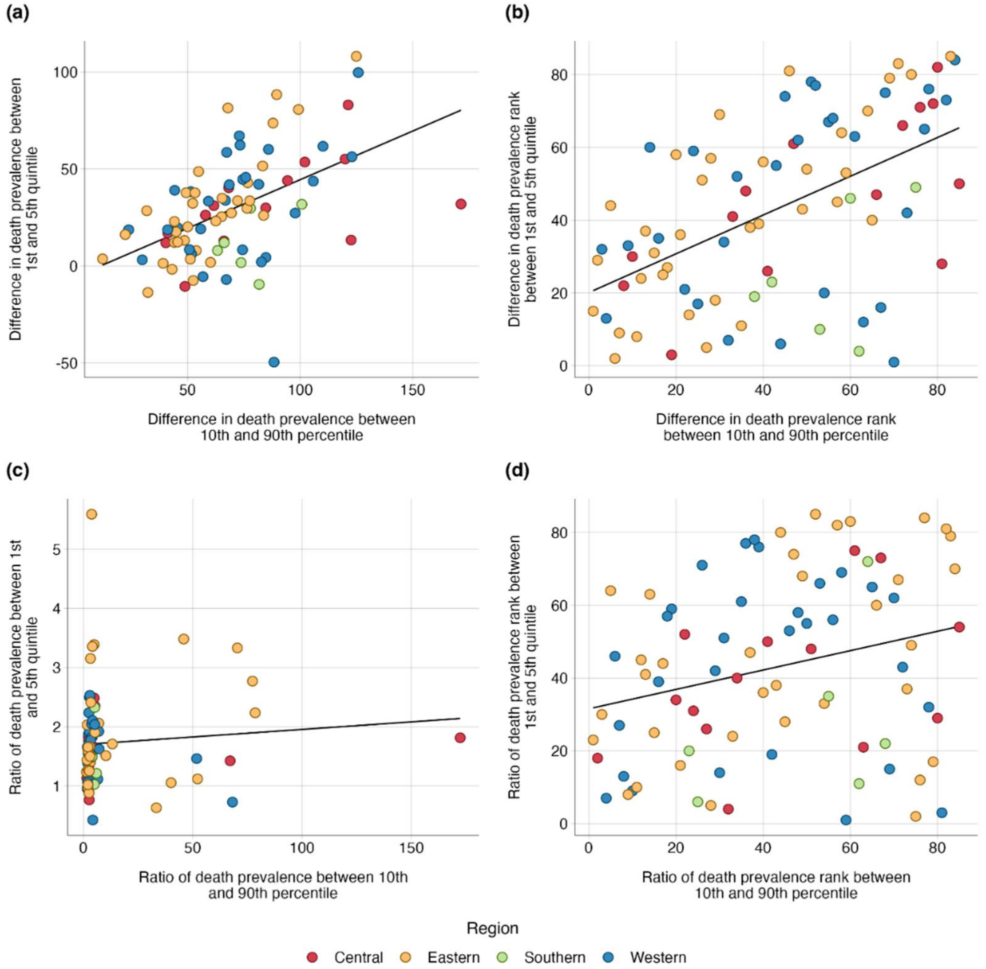

(a) Difference in the under-five death prevalence between the 1st quintile of wealth level (poorest) and the 5th quintile of wealth level (richest) vs. difference in the under-five death prevalence between the 90th percentile and the 10th percentile.

(b) Rank (across all country-years) in the difference in the under-five death prevalence between the 1st quintile of wealth level (poorest) and the 5th quintile of wealth level (richest) vs. rank (across all country years) in the difference in the under-five death prevalence between the 90th percentile and the 10th percentile.

(c) Ratio of the under-five death prevalence of the 1st quintile of wealth level (poorest) divided the 5th quintile of wealth level (richest) vs. ratio in the under-five death prevalence of the 90th percentile divided by the 10th percentile.

(d) Rank (across all country-years) in the ratio of the under-five death prevalence of the 1st quintile of wealth level (poorest) divided by the 5th quintile of wealth level (richest) vs. rank (across all country-years) in the ratio of the under-five death prevalence of the 90th percentile divided by the 10th percentile.

Countries are colored by subregion, defined by the United Nations geoscheme as follows:

Central: Angola, Cameroon, Chad, Congo, Democratic Republic of the Congo, Gabon; Eastern: Burundi, Ethiopia, Kenya, Madagascar, Malawi, Mozambique, Rwanda, Tanzania, Uganda, Zambia, Zimbabwe; Southern: Lesotho, Namibia; Western: Benin, Burkina Faso, Ghana, Guinea, Liberia, Mali, Niger, Nigeria, Senegal, Sierra Leone, Togo.

Table S1. List of household Demographic and Health Surveys and Malaria Indicator Surveys and corresponding sub-Saharan African countries and years used in the analysis. Summary indicators of under-five mortality rate (extracted from the surveys) and gross domestic product (GDP) per capita (extracted from the World Bank's World Development Indicators database) are indicated.

Country years	GDP per capita (USD)	Under-five mortality (per 1,000 live births)		
		Quintile I	Quintile V	Total
Angola				
2015-16	3506	102	39	68
2011	3588	107	83	91
2006-07	2600	-	-	-
Benin				
2017-18	1137	108	60	96
2011-12	1130	88	44	70
2006	855	151	83	125
2001	518	198	93	160
Burkina Faso				
2017-18	738	-	-	-
2010	648	175	97	129
2003	374	206	144	184
Burundi				
2016-17	282	119	52	78
2012	252	-	-	-
2010	234	152	80	96
Cameroon				
2018	1534	111	49	80
2011	1405	184	72	122
2004	1010	189	88	144
Chad				
2014-15	1020	161	138	133
2004	454	176	187	191
Congo				
2011-12	2828	89	54	68
2005	2571	135	85	117
Congo, Democratic Republic of				
2013-14	458	117	76	104
2007	286	184	97	148
Ethiopia				
2016	717	90	67	67
2011	354	137	86	88
2005	162	130	92	123
2000	124	152	80	166
Gabon				
2012	9259	75	50	65
2000	10170	93	55	89
Ghana				
2019	2202	-	-	-

2014	1971	92	64	60
2008	1211	103	60	80
2003	368	128	88	111
Guinea				
2018	983	133	44	111
2012	717	173	68	123
2005	322	217	113	163
Kenya				
2015	1337	-	-	-
2008-09	902	98	68	74
2003	430	149	91	115
Lesotho				
2014	1223	77	70	85
2009	891	107	80	117
2004	753	114	82	113
Liberia				
2016	715	-	-	-
2011	597	-	-	112
2007	397	138	117	110
Madagascar				
2016	476	-	-	-
2011	531	-	-	-
2008-09	536	106	48	72
Malawi				
2017	357	-	-	-
2010	479	133	105	112
2000	156	231	149	189
Mali				
2018	831	143	57	101
2012-13	779	112	61	95
2006	523	233	124	191
Mozambique				
2018	499	-	-	-
2011	595	129	91	97
2003	324	196	108	152
Namibia				
2013	5378	67	31	54
2006-07	3975	92	29	69
2000	2136	63	29	62
Niger				
2012	526	144	114	127
2006	335	206	157	198
Nigeria				
2018	2033	173	53	132
2010	2292	193	78	143
2003	795	257	79	201
Rwanda				
2017	772	-	-	-

2010	612	119	75	76
2000	219	228	154	196
Senegal				
2019	1447	72	27	37
2012-13	1330	90	42	65
2005	994	183	64	121
Sierra Leone				
2016	501	-	-	-
2013	717	186	144	156
2008	408	211	144	140
Tanzania				
2017	1005	-	-	-
2010	743	103	84	81
2004-05	459	137	93	112
Togo				
2017	626	-	-	-
2013-14	621	120	46	88
Uganda				
2018-19	767	-	-	-
2009	793	-	-	86
2000-01	262	192	106	151
Zambia				
2018	1556	67	57	61
2013-14	1879	100	58	75
2007	1124	124	110	119
2001-02	382	192	92	168
Zimbabwe				
2015	1445	102	52	69
2010-11	948	85	58	84
2005-06	477	72	57	82

Notes: Data are from survey reports. "-" indicates that under-five mortality was not reported in the survey for that year.

Table S2. Percentiles in the probability density (full) distribution of under-five death prevalence (per 1,000), since 2000 for 30 sub-Saharan African countries.

Country	Year	10%	20%	30%	40%	50%	60%	70%	80%	90%
Angola	2006	0	29	38	51	57	70	93	130	171
Angola	2011	30	39	57	61	65	69	78	85	94
Angola	2016	26	32	40	45	49	54	59	62	66
Benin	2001	39	55	83	97	105	115	122	140	159
Benin	2006	53	66	74	77	84	92	98	100	111
Benin	2011	32	36	43	47	54	57	59	64	70
Benin	2017	44	49	55	59	67	77	78	83	86
Burkina Faso	2003	71	80	95	102	115	121	133	139	145
Burkina Faso	2010	47	57	63	70	82	86	91	113	133
Burkina Faso	2017	0	12	15	17	19	21	26	29	36
Burundi	2010	30	40	43	61	64	67	69	91	92
Burundi	2012	0	6	14	22	34	44	54	67	176
Burundi	2016	24	27	34	35	47	52	56	58	60
Cameroon	2010	68	72	78	94	97	109	114	122	140
Cameroon	2011	38	49	63	72	76	88	93	105	132
Cameroon	2018	34	49	52	56	63	64	69	78	86
Chad	2004	80	85	109	119	124	144	153	160	171
Chad	2014	77	79	80	88	88	94	101	106	110
Congo	2005	29	45	71	75	84	105	115	124	140
Congo	2011	32	38	44	48	51	54	54	68	81
DRC	2007	36	62	76	78	102	116	129	135	176
DRC	2013	51	61	68	74	74	80	80	84	104
Ethiopia	2000	39	69	99	112	123	125	126	129	131
Ethiopia	2005	19	26	61	65	72	75	83	106	121
Ethiopia	2011	34	55	61	61	65	68	70	77	79
Ethiopia	2016	22	44	49	50	50	58	59	66	68
Gabon	2000	51	58	61	62	65	73	78	84	94
Gabon	2012	15	32	45	46	51	57	65	69	71
Ghana	2003	35	57	62	70	83	92	94	96	114
Ghana	2008	13	33	40	53	65	75	82	102	117
Ghana	2014	15	29	35	42	50	54	60	63	73
Ghana	2019	0	0	0	15	21	23	33	45	54
Guinea	2005	78	83	94	97	111	119	121	146	148
Guinea	2012	36	51	53	69	83	85	90	107	117
Guinea	2018	21	43	56	61	75	87	96	105	129
Kenya	2003	29	50	65	76	85	93	96	103	117
Kenya	2008	33	37	46	50	54	66	74	82	93
Kenya	2015	0	0	14	21	26	36	43	54	60
Lesotho	2004	76	87	88	96	97	106	109	111	139
Lesotho	2009	43	65	72	83	95	104	108	111	121
Lesotho	2014	20	22	51	61	70	73	82	94	108
Liberia	2007	65	65	71	79	83	84	88	91	93
Liberia	2011	32	37	44	48	50	51	59	83	92
Liberia	2016	0	0	22	31	43	50	60	69	95
Madagascar	2008	22	32	45	48	49	57	62	65	71
Madagascar	2011	8	14	21	23	24	26	27	36	42
Madagascar	2016	0	0	5	7	8	9	10	11	13
Malawi	2000	97	113	114	119	121	124	143	148	167
Malawi	2010	63	72	73	76	80	82	84	86	90
Malawi	2017	0	0	0	0	15	19	23	26	52
Mali	2006	69	83	101	110	119	125	142	145	154
Mali	2012	33	42	49	64	67	68	79	87	95

Mali	2018	28	44	50	61	66	76	80	88	93
Mozambique	2003	73	92	96	105	112	113	122	127	131
Mozambique	2011	54	55	59	63	69	75	83	87	95
Mozambique	2018	0	15	23	24	31	33	39	44	53
Namibia	2000	0	24	30	38	49	56	63	69	92
Namibia	2006	29	39	48	48	55	63	66	72	83
Namibia	2013	11	21	32	34	44	53	62	70	78
Niger	2006	47	54	62	76	77	92	101	121	185
Niger	2012	38	48	51	68	70	77	83	86	103
Nigeria	2003	47	91	103	119	120	137	158	179	186
Nigeria	2010	44	56	72	76	94	103	111	126	153
Nigeria	2018	55	62	73	81	90	96	104	112	118
Rwanda	2000	58	76	92	97	129	138	141	147	152
Rwanda	2010	19	30	36	48	55	56	59	72	109
Rwanda	2017	0	10	13	17	18	20	23	34	53
Senegal	2005	42	51	55	61	71	82	84	86	96
Senegal	2012	18	29	36	39	44	46	52	57	70
Senegal	2019	14	21	27	33	36	39	42	57	60
Sierra Leone	2008	70	85	98	100	104	111	119	129	153
Sierra Leone	2013	94	100	101	111	112	116	125	131	138
Sierra Leone	2016	16	23	25	27	30	37	44	66	83
Tanzania	2004	39	48	63	69	80	86	93	97	114
Tanzania	2010	42	49	51	58	62	64	73	78	86
Tanzania	2017	0	14	19	21	23	29	32	52	65
Togo	2013	22	38	49	53	56	66	74	85	93
Togo	2017	0	0	0	12	22	31	34	38	45
Uganda	2000	42	68	73	88	99	109	115	133	153
Uganda	2009	35	49	57	58	65	71	73	76	99
Uganda	2018	9	17	18	21	26	30	37	40	42
Zambia	2002	77	89	92	112	125	132	136	147	160
Zambia	2007	54	69	76	77	80	89	90	103	112
Zambia	2013	33	41	46	50	51	55	56	59	80
Zambia	2018	13	33	38	41	44	49	54	58	66
Zimbabwe	2005	37	50	56	62	64	71	75	88	104
Zimbabwe	2010	25	43	58	62	63	66	84	95	100
Zimbabwe	2015	18	31	38	45	53	55	68	72	77

Notes: Some countries for a certain survey year might have zero mortality due to small sample size.

Table S3. Number of clusters included in each country-year Demographic and Health Survey and Malaria Indicator Survey.

Country	Year	Number of clusters
Angola	2006	36
Angola	2011	39
Angola	2016	38
Benin	2001	27
Benin	2006	32
Benin	2011	34
Benin	2017	35
Burkina Faso	2003	20
Burkina Faso	2010	20
Burkina Faso	2017	25
Burundi	2010	13
Burundi	2012	15
Burundi	2016	12
Cameroon	2010	34
Cameroon	2011	33
Cameroon	2018	34
Chad	2004	24
Chad	2014	21
Congo	2005	27
Congo	2011	24
DRC	2007	17
DRC	2013	17
Ethiopia	2000	10
Ethiopia	2005	14
Ethiopia	2011	17
Ethiopia	2016	17
Gabon	2000	25
Gabon	2012	22
Ghana	2003	31
Ghana	2008	38
Ghana	2014	44
Ghana	2019	47
Guinea	2005	24
Guinea	2012	29
Guinea	2018	34
Kenya	2003	28
Kenya	2008	31
Kenya	2015	33
Lesotho	2004	16
Lesotho	2009	20
Lesotho	2014	23
Liberia	2007	13
Liberia	2011	14
Liberia	2016	22
Madagascar	2008	19
Madagascar	2011	19

Madagascar	2016	23
Malawi	2000	13
Malawi	2010	17
Malawi	2017	18
Mali	2006	30
Mali	2012	30
Mali	2018	37
Mozambique	2003	18
Mozambique	2011	27
Mozambique	2018	33
Namibia	2000	29
Namibia	2006	27
Namibia	2013	29
Niger	2006	20
Niger	2012	21
Nigeria	2003	39
Nigeria	2010	40
Nigeria	2018	40
Rwanda	2000	14
Rwanda	2010	13
Rwanda	2017	18
Senegal	2005	32
Senegal	2012	33
Senegal	2019	39
Sierra Leone	2008	20
Sierra Leone	2013	19
Sierra Leone	2016	19
Tanzania	2004	19
Tanzania	2010	22
Tanzania	2017	34
Togo	2013	34
Togo	2017	36
Uganda	2000	19
Uganda	2009	19
Uganda	2018	26
Zambia	2002	19
Zambia	2007	27
Zambia	2013	28
Zambia	2018	25
Zimbabwe	2005	25
Zimbabwe	2010	29
Zimbabwe	2015	33
